# Pre-analytical stability of the plasma proteomes based on the storage temperature

**DOI:** 10.1186/1477-5956-11-10

**Published:** 2013-03-21

**Authors:** Sara Pasella, Angela Baralla, Elisabetta Canu, Sara Pinna, James Vaupel, Marta Deiana, Claudio Franceschi, Giovannella Baggio, Angelo Zinellu, Salvatore Sotgia, Giuseppe Castaldo, Ciriaco Carru, Luca Deiana

**Affiliations:** 1 Biochimica Clinica e Biologia Molecolare Clinica, Dipartimento di Scienze Biomediche, Università di Sassari, Sassari, Italy; 2 Max Planck Institute for Demographic Research, Rostock, Germany; 3 Associazione “Isola dei Centenari”, Sassari, Italy; 4 Dipartimento di Patologia Sperimentale, Università di Bologna, Bologna, Italy; 5 Azienda Ospedaliera di Padova, Padua, Italy; 6 Dipartimento di Biochimica e Biotecnologie Mediche, Universita’ di Napoli Federico II, Naples, Italy; 7 Azienda Ospedaliera Universitaria di Sassari, Sassari, Italy; 8 Center for Biotechnology Development and Biodiversity Research, University of Sassari, Sassari, Italy

**Keywords:** Two-dimensional electrophoresis, Mass spectrometry, Plasma proteome, Specimen collection and handling, Storage temperature

## Abstract

**Background:**

This study examined the effect of storage temperature on the protein profile of human plasma. Plasma samples were stored for 13 days at -80°C, -20°C, +4°C and room temperature (20-25°C) prior to proteomic analysis. The proteomic comparisons were based on the differences of mean intensity values of protein spots between fresh plasma samples (named “time zero”) and plasma samples stored at different temperatures. To better understand the thermally induced biochemical changes that may affect plasma proteins during storage we identified proteins with different expressions with respect to the time zero sample.

**Results:**

Using two-dimensional electrophoresis followed by MALDI-TOF MS and /or LC-MS/MS 20 protein spots representing 10 proteins were identified with significant differences in abundance when stored at different temperatures. Our results, in agreement with various authors, indicate that during storage for a short period (13 days) at four different temperatures plasma proteins were more affected by degradation processes at +4°C compared to the other temperatures analysed. However, we founded that numerous protein spots (vitamin D binding protein, alpha-1-antitrypsin, serotransferrin, apoplipoprotein A-I, apolipoprotein E, haptoglobin and complement factor B) decrease in abundance with increasing temperature up to 4°C, but at room temperature their intensity mean values are similar to those of time zero and -80°C. We hypothesize that these proteins are labile at 4°C, but at the same time they are stable at room temperature (20-25°C). Furthermore we have grouped the proteins based on their different sensitivity to the storage temperature. Spots of serum albumin, fibrinogen gamma chain and haptoglobin are more resistant to the higher temperatures tested, as they have undergone changes in abundance only at room temperature; conversely, other spots of serum albumin, fibrinogen beta chain and serotransferrin are more labile as they have undergone changes in abundance at all temperatures except at -80°C.

**Conclusions:**

Although there are many studies concerning protein stability of clinical samples during storage these findings may help to provide a better understanding of the changes of proteins induced by storage temperature.

## Background

Human serum and plasma are the favored specimens and they are easily accessible for many analysis in proteome research, especially in the biomarker discovery field [1]. The human blood proteome has a great biological and clinical importance and reflects the environment of all cells, tissues and organs in the human body. Human serum and plasma contains several different water-soluble components, among them the plasma/serum proteins. In principle, any protein can become at least temporarily a blood plasma protein depending on the actual state of the body, e.g. especially in the case of a pathological situation [2]. Moreover, a protein that is not usually present in blood plasma can accumulate in it as a characteristic diagnostic marker for a certain disease [3]. Due to the continuous development of proteomic techniques mainly centered around mass spectrometric identification [4], the number of proteins annotated and identified in plasma/serum in recent years is considerably increased. However proteomic investigations involving human serum and plasma are complicated because there are a wide range of pre-analytical variables that could lead to alteration of the detected proteome [5]. The more important variables for sampling procedures of plasma/serum proteomes are related to the selection of blood collection tubes and anticoagulants [6-8], variations in clotting time or time lag before centrifugation [9], hemolysis, centrifugation speed or time, storage temperature [10] and repeated freeze/thaw cycles [9,11]. Moreover, in proteomic research it’s very important the choice of the sample type [7,12]; the quality of specimens affects the validity of the analytical data, which makes the integrity of samples an important issue. In principle, depending on analytical objectives and/or target peptides or proteins, the use of either plasma or serum may impact both method and results. In regard to peptidomics, Tammen et al. [13] found that a significant number of peptides was different not only between serum and plasma specimens but also among plasma specimens prepared with different anticoagulants. Plasma and serum protein profiles reported by various laboratories are often variable, reflecting different analytical methods and sample preparation processes [11,13-15]. In this context it is very important to establish a standard protocol to monitor the stability of human plasma/serum samples upon storage. In this work we studied the pre-analytical stability of the plasma proteomes based on the storage temperature using proteomic techniques including two dimensional electrophoresis and MALDI-TOF MS (matrix assisted laser desorption/ionization time of-flight mass spectrometry) and/or LC-MS/MS (liquid chromatography-tandem mass spectrometry) [16-18]. Plasma samples were stored for 13 days at -80°C, -20°C, +4°C and room temperature (20-25°C) prior to proteomic analysis. The proteomic comparisons were based on the differences of the mean intensity values of the protein spots between fresh plasma samples (named “time zero”) and plasma samples stored at different temperatures. To better understand the thermally induced biochemical changes that may affect plasma proteins during storage we identified proteins with different expressions with respect to the time zero sample. The subjects examined were recruited through the “AkeA Project” [19] (approved by the bioethical committee) that study human longevity in Sardinia and includes a very great number of plasma samples of Sardinian people spanning from 20 to 112 years. In this project, started in 1997, the researcher’s group performed good results in the collection procedures, in the study of the biochemical, genetic and environmental conditions involved in the ageing processes. Moreover, within the “AkeA project” proteomic studies were performed in order to evaluate changes in proteins associated with human longevity.

## Results and discussion

**Figure 1:**
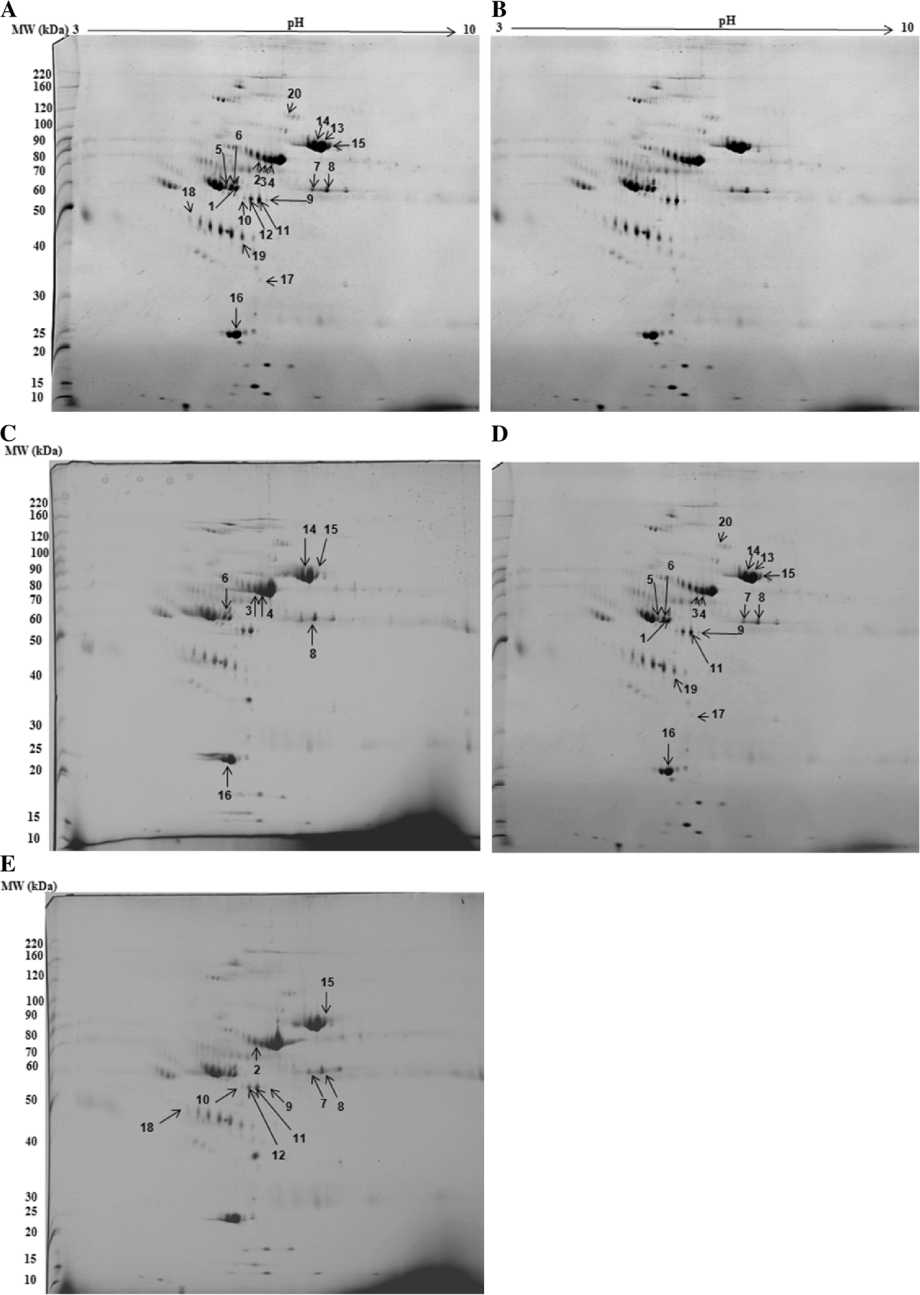
**Images from the 2D gels obtained in this work. **(**A**) 2-DE gel image with plasma proteins of control (time zero, fresh plasma samples); (**B**-**E**) 2-DE gel images corresponding to the plasma samples stored at -80°C, -20°C, +4°C and room temperature respectively. The plasma was separated on 2-DE gels and visualized by Coomassie blue staining. Proteins undergoing a significant variation in abundance (>2-fold change) are numbered and indicated by arrows. The molecular weights (MW) and pI scales are indicated. Each gel is representative of three independent replicates.

**Figure 2:**
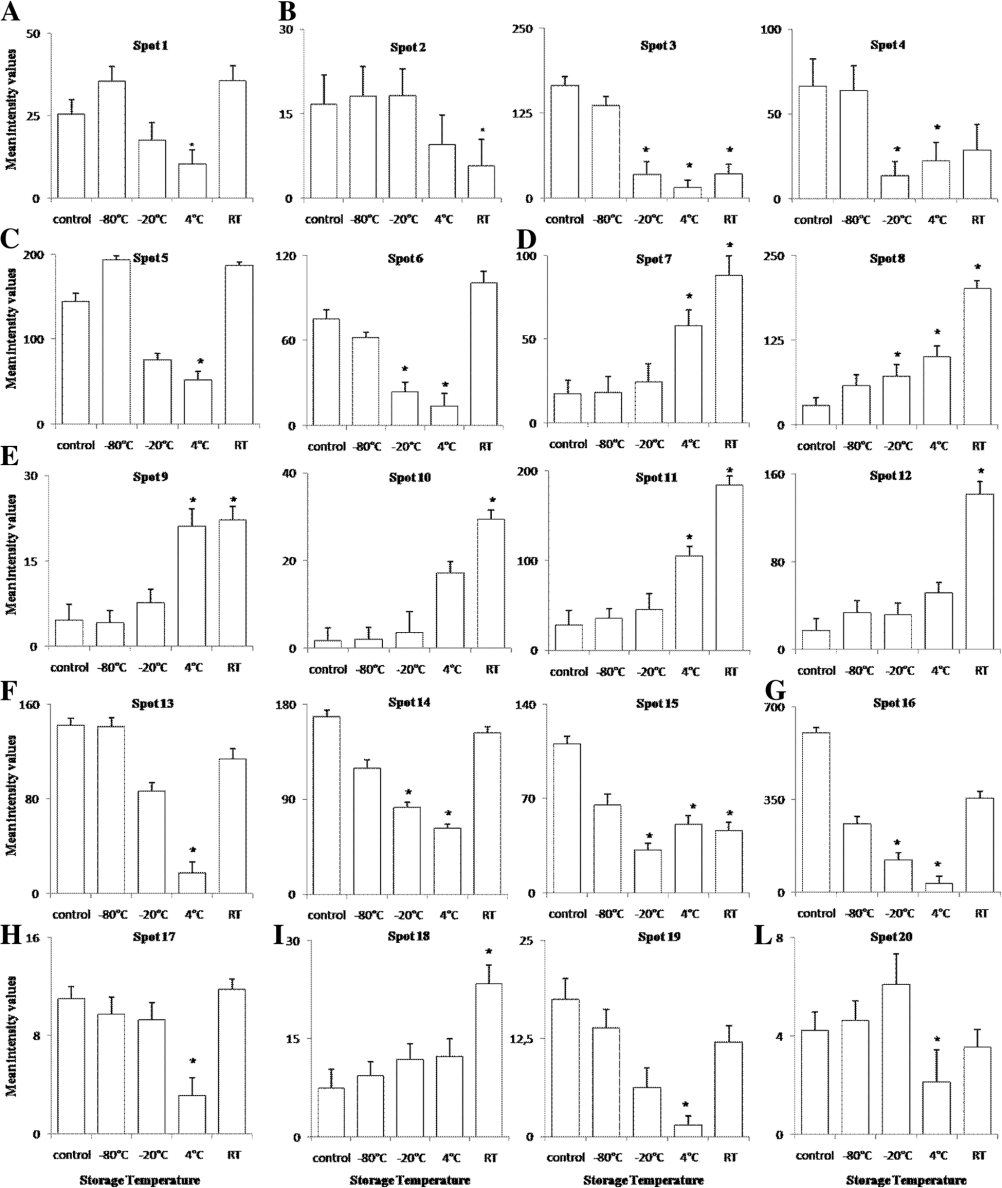
**Protein patterns showing significant changes (>2-fold) under different storage temperature with respect to time zero. **The letters A-L represent the protein spots that showed statistically significant (p ≤ 0.05) change from control (time zero, fresh plasma samples): (**A**) vitamin D-binding protein; (**B**) serum albumin; (**C**) alpha-1-antitrypsin; (**D**) fibrinogen beta chain; (**E**) fibrinogen gamma chain; (**F**) serotransferrin; (**G**) apolipoprotein A-I; (**H**) apolipoprotein-E; (**I**) haptoglobin; (**L**) Complement factor B. The data represent the mean intensity values (±SD) from three replicates at each temperature. The bars with an asterisk indicate significant difference (p ≤ 0.05) with respect to the group “time zero”.

In the present work, the influence of the storage temperature on the stability of human plasma proteins was analysed by two-dimensional electrophoresis on polyacrylamide gels (2D-PAGE) and mass spectrometry. The proteomic comparisons were based on the differences of mean intensity values of protein spots between fresh plasma samples (named “time zero”) and plasma samples stored at four different temperatures [-80°C, -20°C, +4°C and room temperature (20-25°C)] for a period of 13 days. The software used for image analysis (PD QUEST) has detected about 500 valid spots of which 20 spots were found to show significant changes (higher than two fold, p ≤ 0.05) in intensities compared with the time zero sample. These spots were further identified by MALDI-TOF MS (Table 1) (see also Additional file 1: Table S1) and/or LC-MS/MS analysis (Table 2) (see also Additional file 2: Table S2). Representative 2D gels image is reported in Figure 1. In general, the 2-D profile appeared well preserved after 13 days of storage at -80°C, in fact, in these the time zero sample was observed. As expected, a higher number of spots showed a significant quantitative variation in plasma stored at higher temperatures. Table 3 shows the 7 spots that significantly change between storage at -20°C and the time zero. Between them we can recognize some of the most abundant proteins in plasma, like serum albumin (3, 4) [Swiss-Prot: P02768], alpha-1-antitrypsin (6) [Swiss-Prot: P01009], fibrinogen beta chain (8) [Swiss-Prot: P02675], serotransferrin (14, 15) [Swiss-Prot: P02787] and apolipoprotein A-I (16) [Swiss-Prot: P02647] with all of them decreasing in intensity if compared to time zero except for the fibrinogen beta chain. Table 4 shows the 16 spots that change during storage at 4°C compared to time zero; the protein spots comprise all of the ones that change with storage at -20°C and 9 more spots: Vitamin D-binding protein (1) [Swiss-Prot: P02774], Alpha-1-antitrypsin (5), Fibrinogen beta chain (7), Fibrinogen gamma chain (9, 11), Serotransferrin (13), Apolipoprotein-E (17) [Swiss-Prot: P02649], Haptoglobin (19) [Swiss-Prot: P00738] and Complement factor B (20) [Swiss-Prot: P00751]. The majority of these spots decrease in intensity compared to time zero except for fibrinogen beta chain (7, 8) and fibrinogen gamma chain (9, 11) that conversely increase. The Table 5 shows the changes between room temperature and time zero with changes for 10 spots, three of which decrease [albumin (2, 3) and serotransferrin (15)] and nine increase [fibrinogen beta chain (7, 8), fibrinogen gamma chain (9, 10, 11, 12) and haptoglobin (18)]. As seen from the data presented in Figure 2 we observed with interest that most of the altered protein spots decrease in abundance with increasing temperature up to 4°C, but at room temperature their intensity mean values are similar to those of time zero and -80°C. Of 20 spots that were found to show significant changes in intensities with respect to time zero, six spots identified as vitamin D-binding protein (1) [Swiss-Prot: P02774], alpha-1-antitrypsin (5), serotransferrin(13), apolipoprotein E (17) [Swiss-Prot: P02649], haptoglobin (19) [Swiss-Prot: P00738] and complement factor B (20) [Swiss-Prot: P00751] showed a significant abundance decrease only in plasma stored at +4°C with respect to the time zero sample but not showed any statistically significant change at other temperatures. Serotransferrin and haptoglobin (spots 13 and 19 respectively) showed the highest abundance decrease, with an average ratio of 0.12 and 0.09 respectively with respect to time zero (p = 0.016 and 0.038 respectively), whereas the other spots (5, 17 and 20) showed an abundance decrease at the same conditions (Table 4). Among these proteins, spots 1, 5 and 20 showed a higher molecular weight and a lower isoelectric point than their theoretical values. While, spots 3, 17, and 19 showed lower molecular weights and isoelectric points than their theoretical values (Table 1). Moreover, four spots, identified as serum albumin (2), fibrinogen gamma chain (10 and 12) and haptoglobin (18) showed a significant abundance change only in plasma stored at room temperature with respect to the time zero sample but not showed any statistically significant change at the other temperatures. Spots of fibrinogen gamma chain (10 and 12) showed the highest abundance increase with an average ratio of 16.35 and 8.34 respectively with respect to time zero (p = 0.014 and 0.02 respectively), whereas the others spots (2 and 18) showed an average ratio of 0.35 (p = 0.031) and 3.15 (p = 0.047) respectively (Table 5). Various scientists recommend that plasma samples should be stored in liquid nitrogen or at -80°C if this is not available [20]. Sen-Yung Hsieh et al. [11] reported that storage at room temperature (25°C) and at 4°C caused prominent changes in the serum/plasma proteome only after 8 h and 48 h of incubation respectively, particularly for proteins of less than 3000 Da as demonstrated by using magnetic bead-based MALDI-TOF MS. They have also compared serum proteomes from the serum samples freshly obtained or stored at -80°C for 1 and 3 months, respectively and only minimal changes were found. Similar results were reported also by Rai et al. [14] and Marshall et al. [21]. In agreement with mentioned authors, we found that storage temperatures have important effects on plasma protein profiles and that storage of plasma at low temperatures preserves more plasma proteins than storing them at higher temperatures. In summary the protein spots undergoing significant variations in abundance (>2-fold change; p ≤ 0.05) after 13 days of storage at different temperatures with respect to the time zero can be grouped into six main protein families: ALB/AFP/VDB family, serpin family, fibrinogen alpha/beta family, transferrin family, apolipoprotein A-I/A4/E family and peptidase S1 family (Table 1). All the spots undergoing significant variations in abundance with increasing storage temperature showed modest changes of their molecular weights and isoelectric points compared to the native protein. We founded that all protein spots belonging to the fibrinogen alpha/beta family and one spot (18) belonging to the peptidase S1 family undergo a linear increase in abundance with increasing temperature. The apparent increase in the abundance of a spot or a shift in its isoelectric point in the 2-D map can depend on protein modifications occurring as a result of biochemical processes, such as dephosphorylation, oxidation or loss of charged amino acid side chains. Moreover, we founded that most of the protein spots belonging to the other protein families mentioned above, undergo a decrease in abundance up to 4°C, but when storing them at room temperature their intensity mean values are similar to those of the time zero sample. This is the case of proteins identified as vitamin D binding protein (1), alpha-1-antitrypsin (4 and 5), serotransferrin (13 and 14), apoplipoprotein A-I (16), apolipoprotein E (17), haptoglobin (19) and complement factor B (20). The abundance decrease and the shift in the isoelectric points and in the molecular weights of these spots compared to the native protein may be caused by proteolytic processes, leading to the formation of fragments of the native protein. We hypothesize that these proteins are labile at 4°C, but at the same time they are stable at room temperature (20-25°C). Furthermore we have grouped the proteins based on their different sensitivity to the storage temperature; proteins identified as serum albumin (2), fibrinogen gamma chain (10 and 12) and haptoglobin (18) are more resistant to higher temperatures, as they have undergone changes in abundance only to room temperature. Conversely, spot 3 of serum albumin, spot 8 of fibrinogen beta chain and spot 15 of serotransferrin are more labile as they have undergone changes in abundance at all temperatures except at -80°C. Monitoring changes of the protein spots abundance was useful to get an overview of the importance of the degradation processes that occur in plasma samples during the storage period and to identify which proteins are involved in these changes. Although there are many studies concerning protein stability of clinical samples during storage these findings may help to provide a better understanding of the changes of proteins induced by storage temperature.

**Table 1:** Plasma protein spots identified by MALDI-TOF MS

**Spot**	**Identified protein**	**Acc. no. **^**a**^	**Score **^**b**^	**QM **^**c**^	**% C **^**d**^	**Theoretical *M*r/p*I ***^**e**^	**Observed *M*r/p*I ***^**f**^
		**ALB/AFP/VDB family**					
**1**	Vitamin D-binding protein	P02774	100	11	43	52964/5.4	55700/5.3
**2**	Serum albumin	P02768	95	12	28	69367/5.92	67600/5.4
**3**	Serum albumin	P02768	142	18	41	69367/5.92	57000/5.4
**4**	Serum albumin	P02768	224	21	43	69367/5.92	56900/5.6
		**Serpin family**					
**5**	Alpha-1-antitrypsin	P01009	133	17	34	46737/5.37	56000/5.0
**6**	Alpha-1-antitrypsin	P01009	117	10	32	46737/5.37	56100/5.1
		**Fibrinogen alpha/beta family**					
**7**	Fibrinogen beta chain	P02675	41	7	20	55928/8.54	55700/6.3
**8**	Fibrinogen beta chain	P02675	95	10	27	55928/8.54	55300/6.4
**9**	Fibrinogen gamma chain	P02679	177	14	43	51512/5.37	51500/5.7
**10**	Fibrinogen gamma chain	P02679	177	14	45	51512/5.37	51700/5.3
**11**	Fibrinogen gamma chain	P02679	164	14	39	51512/5.37	51200/5.6
**12**	Fibrinogen gamma chain	P02679	177	14	43	51512/5.37	51700/5.5
		**Transferrin family**					
**13**	Serotransferrin	P02787	423	40	54	77064/6.81	57200/6.4
**14**	Serotransferrin	P02787	81	10	20	77064/6.81	57300/6.3
**15**	Serotransferrin	P02787	196	20	31	77064/6.81	57400/6.4
		**Apolipoprotein AI/A4/E family**					
**16**	Apolipoprotein A-I	P02647	60	7	28	30778/5.56	23000/5.2
**17**	Apolipoprotein-E	P02649	151	17	55	36154/5.65	33500/5.6
		**Peptidase S1 family**					
**18**	Haptoglobin	P00738	64	9	19	45205/6.13	45300/5.0
**19**	Haptoglobin	P00738	70	10	25	45205/6.13	42800/5.5
**20**	Complement factor B	P00751	60	5	13	85533/6.67	97900/6.2

^a ^Accession number (database: UniProtKB/Swiss-Prot).

^b ^Score: probability score in MASCOT program (the probability that the observed match between the experimental data and mass values calculated from a candidate peptide sequence is a random event).

^c ^Queries matched indicates the number of matched peptides in the database search.

^d ^Percent coverage: the minimum coverage of the matched peptide in relation to the full-length sequence.

^e ^Theoretical molecular weight/isoelectric point of full-length protein according to the Swiss-Prot database.

^f ^Molecular weight/isoelectric point of full-length protein calculated upon calibration of electrophoretic gels.

**Table 2:** Plasma protein spots identified by LC-MS/MS

**Spot**	**Identified protein**	**Acc. no. **^**a**^	**Score **^**b**^	**QM **^**c**^	**% C **^**d**^
1	Vitamin D-binding protein	P02774	279	24	44
13	Serotransferrin	P02787	441	45	42
17	Apolipoprotein-E	P02649	239	15	39

^a ^Accession number (database: UniProtKB/Swiss-Prot).

^b ^Score: probability score in MASCOT program (the probability that the observed match between the experimental data and mass values calculated from a candidate peptide sequence is a random event).

^c ^Queries matched indicates the number of matched peptides in the database search.

^d ^Percent coverage: the minimum coverage of the matched peptide in relation to the full-length sequence.

**Table 3:** Protein spots undergoing significant changes (>2-fold) after storage at -20°C with respect to time zero

**Spot**	**Protein name**	**Average ratio -20°C/time zero**	**Paired t-test**
3	Serum albumin	0.20	0.047
4	Serum albumin	0.20	0.022
6	Alpha-1-antitrypsin	0.31	0.016
8	Fibrinogen beta chain	2.51	0.046
14	Serotransferrin	0.49	0.007
15	Serotransferrin	0.29	0.016
16	Apolipoprotein A-I	0.20	0.039

**Table 4:** Protein spots undergoing significant changes (>2-fold) after storage at +4°C with respect to time zero

**Spot**	**Protein name**	**Average ratio - +4°C/time zero**	**Paired t-test**
1	Vitamin D-binding protein	0.40	0.02
3	Serum albumin	0.09	0.047
4	Serum albumin	0.33	0.05
5	Alpha-1-antitrypsin	0.36	0.016
6	Alpha-1-antitrypsin	0.18	0.016
7	Fibrinogen beta chain	3.27	<0.001
8	Fibrinogen beta chain	3.50	0.031
9	Fibrinogen gamma chain	4.59	0.006
11	Fibrinogen gamma chain	3.70	0.035
13	Serotransferrin	0.12	0.016
14	Serotransferrin	0.37	0.008
15	Serotransferrin	0.46	0.037
16	Apolipoprotein A-I	0.05	0.02
17	Apolipoprotein-E	0.28	0.002
19	Haptoglobin	0.09	0.038
20	Complement factor B	0.50	0.015

**Table 5:** Protein spots undergoing significant changes (>2-fold) after storage at RT with respect to time zero

**Spot**	**Protein name**	**Average ratio -RT/time zero**	**Paired t-test**
2	Serum albumin	0.35	0.031
3	Serum albumin	0.21	0.047
7	Fibrinogen beta chain	4.98	<0.001
8	Fibrinogen beta chain	7.06	0.002
9	Fibrinogen gamma chain	4.83	<0.001
10	Fibrinogen gamma chain	16.35	0.014
11	Fibrinogen gamma chain	6.49	0.006
12	Fibrinogen gamma chain	8.34	0.02
15	Serotransferrin	0.42	0.037
18	Haptoglobin	3.15	0.047

## Conclusions

In agreement with various authors, the comparative proteomic analysis of human plasma samples under different storage temperatures indicated that storage at high temperature reduces considerably the stability of plasma proteomes. However, we observed with interest that some proteins are more labile at +4°C than at room temperature. Furthermore we observed modest variations of the molecular weight and isoelectric point of these protein spots compared to the native proteins. Even if more studies are needed, these findings may be useful in understanding the degradative mechanisms taking place in the plasma samples in order to eliminate possible mistakes in the pre-analytical phase due to storage temperature.

## Methods

### Materials

Protease Inhibitor Cocktail for general use, Angiotensina II, [Val5]Ang I, [Glu1]-Fibrinopeptide B human, ACTH [1-17], ACTH [18–39], ACTH [7–38], alpha cyano-4-hydroxycinammico (CHCA), trifluoroacetic acid (TFA) and Acetonitrile LC-MS CHROMASOLV were from Sigma Aldrich.

### Human plasma samples

The population was chosen in order to have a low variability of gender and age. Plasma samples were from 6 subjects including 3 males and 3 females aged between 23 and 32 years. The donors were healthy volunteers and/or not suffering from diseases such as vascular disease, stroke, diabetes or cancer. All volunteers were recruited from the Akea Project (project approved by the local bioethics) and all of them signed a written consent prior to blood sampling. Blood samples were collected early in the morning to reduce the biological variability, by venipuncture into vacutainer (Greiner bio-one, Austria) containing K2 EDTA as anticoagulant. Immediately after the blood collection protease inhibitors (Protease Inhibitor Cocktail, Sigma P2714) were added in the ratio of 1:100 into the vacutainer; then we proceeded to the separation of the plasma by centrifugation at 2500 g for 15 minutes at 4°C. The supernatant containing the plasma was divided into a defined number of aliquots of 200 μl each. The blood and plasma sample handling and processing was carried out in a short time (about 30 min). One aliquot of plasma sample was used for protein extraction in the same day of which blood samples were collected; plasma sample purified were then used for 2DE and MS and/or MS/MS analysis; this sample, named “time zero” was used as a reference sample for all subsequent proteomic comparisons. The other aliquots were stored at -80°C, -20°C, +4°C and room temperature (20 to 25°C) respectively and were analyzed after 13 days. For each experiment studied (time zero, -80°C, -20°C, 4°C and room temperature ) we analyzed a pooled sample comprising equal amounts of each of the samples in the study. As we have considered three replicates for each sample we analyzed a total of 15 gels. Proteins were extracted from plasma samples using Albumin and IgG Depletion kit (ProteoPrep^®^ Blue Albumin and IgG Depletion kit, Sigma, P1120) according to the manufacturer’s instructions [22-24]. The protein extracts were resuspended in sample buffer containing Urea 7 M, Thyourea 2 M and CHAPS 4% and quantified by a modified Lowry assay according to the manufacturer’s instructions (DC protein assay kit, BioRad).

### Two-dimensional gel electrophoresis (2-DE) and visualization

Total protein extracts were separated by 2D-PAGE gels [25]. Analytical gels contained 250 μg of total protein extracts; for preparative gels 1000 μg of protein were applied. Three experimental replicates were performed for each sample. The first dimension isoelectric focusing (IEF) was performed using the Protean IEF Cell system (Biorad). Immobilized pH gradient strips (IPG strips) (linear pH 3–10, 17 cm) were rehydrated with rehydration buffer (urea 8 M, thyourea 2 M, 4% CHAPS, destreak reagent, 10 mM dithiothreitol, 1% carrier ampholyte 5–8 and 0.05% bromophenol blue) for 20 hours at 20°C without voltage. IEF was carried out using a multi-step procedure (2 hr at 250 V, 2 hr at 500 V, 2 hr at 750 V, 2 hr at 1000 V, 2 hr at 5000 V, 2 hr at 8000 V, at 8000 V for 50.000 Volthours, for a total of 70.000 V). Focused IPG strips were equilibrated in two steps (15 min each) in 1 ml freshly prepared sample buffer (50 mM Tris–HCl pH 8.8, 6 M urea, 20% (v/v) glycerol and 2% (w/v) SDS, 1% (w/v) bromophenol blue) supplemented with 2% dithiothreitol and 2.5% iodoacetamide respectively. Equilibrated IPG strips were transferred into 5-15% gradient acrylamide gels and embedded with 0.5% w/v melted agarose. Separation in the second dimension was performed for 15 min at 25 mA and then for ~4 hr at 50 mA in Tris-glycine-SDS running buffer, using the Protean Multicell (BioRad) apparatus [26].

### Image analysis

The gels were stained overnight in Coomassie Brilliant Blue R-250 (CBB R-250) solution (0.05% (w/v) CBB R-250, 50% (v/v) methanol, 10% (v/v) acetic acid). Gels were scanned using a GS-800 densitometer (BioRad) and analyzed with the PDQuest 2D image analysis software (advanced version, BioRad) [27,28]. The image analysis allowed to compare the protein spot mean intensity values (OD, optical density) from the different experiments. The volume of all matched spots were normalized with the “Local Regression Model” method (LOESS); this method uses the raw quantity of each spot in a member gel, multiplied by a factor based on the local regression method of the matched spots. In order to exclude artifacts and false positive we confirmed manually both matching and data quality of all the spots: only those spots that were detectable in all gels of a sample set were considered for evaluation.

### In-gel digestion of proteins

Spots of interest were manually excised from the preparative gels, chopped up into little pieces and digested with trypsin [29]. Each small gel piece with protein was transferred into tubes, washed 3 times for 15 minutes with 50 mM NH_4_HCO_3_ pH 8 and then with100% acetonitrile (ACN) for another 15 minutes. The gel pieces were reduced with 10 mM DTT in 50 mM NH4HCO3 for 45 min at 56°C and alkylated with 55 mM iodoacetamide in 50 mM NH4HCO3 for 30 min at room temperature in the dark. They were washed 5 times for 15 minutes with 50 mM NH_4_HCO_3_ pH 8 and then with 100% ACN for another 15 minutes. After total evaporation of the ACN the gel pieces were hydrolyzed with 10 μl of trypsin (10 ng/ul) in 50 mM NH4HCO3 pH 8. After 2 hours of incubation on ice the supernatant was removed and the gel pieces were resuspended in 50 mM NH4HCO3 pH 8; the samples were incubated overnight at 37°C. After digestion, the protein peptides were acidified with 20% TFA (1 μl TFA 20%/10 μl of protein peptides), evaporated in a vacuum centrifuge and resuspended in 5 μl of 2% TFA.

### Protein identification by mass spectrometry

Protein spots with a statistically significant variation (p ≤ 0.05), showing an over two-fold difference in volume, were selected as differentially expressed and analyzed by MS and MS/MS analysis. MS analysis was performed on a MALDI-TOF mass spectrometer micro MX™(Waters) [30,31]. One μl of tryptic peptide solution of each digested spot was mixed with an equal amount of the matrix α-cyano-4-hydroxycinnamic acid (CHCA), prepared in 0,2% TFAaq and in 70% CAN, applied on a MALDI plate and dried at room temperature. All spectra were acquired in reflectron mode, positive ion, mass range from 800–4000 Da. Ionization was performed by irradiation with a nitrogen laser (wave length, 337 nm) operating at 20 Hz. For matrix suppression, we used an high gating factor with signal suppression of up to 800 Da. The spectra were calibrated externally resulting in mass accuracy better than 100 ppm [32], using the following mixture of peptides: Angiotensina II (m/z 1046,5423 Da), [Val5]Ang I (m/z 1282,662 Da), [Glu1]-Fibrinopeptide B human (m/z1570,67 Da), ACTH [1-17] (m/z 2093,09 Da), ACTH [18–39] (m/z 2465,2 Da), ACTH [7–38] (m/z 3657,93 Da). All spectra were analyzed using the Mass Linx v 4.1 software (Waters). Protein identification was performed by peptide mass fingerprinting (PMF) using MASCOT software searching at http://www.matrixscience.com. Search parameters were restricted to *Homo sapiens* taxonomy using the SwissProt database. Enzyme selection was trypsin, with up to one missed cleavage permitted. Carbamidomethylation of cysteines was selected as a fixed modification; Gln- > pyro-Glu (N-term Q),Oxidation (M) as variable modifications. Protein mass was unrestricted, and peptide mass tolerance typically set at ±150 ppm. Mass values were entered as monoisotopic MH+. LC-MS/MS analysis was performed on an XCT Ultra 6340 ion trap equipped with a 1200 HPLC system and a chip cube (Agilent Technologies, Palo Alto, CA, USA). After loading, samples were concentrated and desalted at 4 mL/min on a 40-nL enrichment column (75 mm_43 mm, Agilent Technologies), with 0.2% formic acid. Peptides were then fractionated on a C18 reverse-phase capillary column at a flow rate of 300 nL/min, with a linear gradient of eluent B (0.2% formic acid in 95% ACN) in A (0.2% formic acid in 2% ACN) from 3–60% in 20 min. ESI parameters were as follows: capillary voltage 1730 V; dry gas (N2), 5.00 L/min; dry temperature, 3251C; trap drive, 100; skimmer 30 V; lens 1, _5.00 V; octopole RF amplitude, 200 Vpp; capillary exit, 90 V. The ion-trap mass spectrometer was operated in a positive-ion mode. Trap ICC smart target was 30 0000 units and maximal accumulation time was 100 ms. MS/MS was operated at a fragmentation amplitude of 1.3 V, and threshold ABS was 6000 units. Scan speed was 8100 UMA/s in MS and 26 000 UMA/s in MS/MS scans. Peptide analysis was performed scanning from m/z 250 to m/z 2200 in AutoMS (n) precursor selection mode of the three most intense ions (fragmentation mass range from 100 to 2200 m/z). Dynamic exclusion was used to acquire a more complete survey of the peptides by automatic recognition and temporary exclusion (0.15 min) of ions from which definitive mass spectral data had previously acquired. Data analysis software, provided by the manufacturers, was used to analyze MS/MS spectra and to generate a peak list that was introduced in the in-house MASCOT MS/MS ion search software (Version 2.3, Matrix Science, Boston, MA, USA) for protein identification in the NCBI database using the Homo sapiens taxonomy. Search parameters were as follows: peptide tolerance 300 ppm, MS/MS tolerance 0.6 Da, enzyme trypsin, allowing one missed cleavage. Carbamidomethylation of cysteines was selected as a fixed modification; Gln- > pyro-Glu (N-term Q),Oxidation (M), Phospho (ST), Phospho (Y) as variable modifications.

### Statistical data analysis

The average intensities of resolved spots were compared using quantitative (2.0-fold increase or decrease ratios) functions within the PDQuest software. The quantity tables were exported and all the statistical analysis were performed using the paired t-test implemented in the Sigma Stat 3.1 software. P-values ≤0.05 were considered significant. Among all statistically significant spots, only those that showed changes of the mean intensity values at least by a factor 2 with respect to the group “time zero” were selected, in order to exclude the impact of experimental variability and consider more reliable data.

## Abbreviations

MALDI-TOF MS: Matrix assisted laser desorption/ionization time of-flight mass spectrometry; LC-MS/MS: (Liquid chromatography-tandem mass spectrometry; 2D-PAGE: Two-dimensional polyacrylamide gel electrophoresis; IEF: Isoelectric focusing; IPG strips: Immobilized pH gradient strips; CBB R-250: Coomassie Brilliant Blue R-250; ACTH: Adrenocorticotropic hormone; CHCA: Alpha cyano-4-hydroxycinammico; ACN: Acetonitrile; TFA: Trifluoroacetic acid; EDTA: Ethylenediaminetetraacetic acid; TCA: Trichloroacetic acid; CHAPS: 3-[(3-cholamidopropyl)dimethylammonio]-1-propanesulfonate; DTT: Dithiothreitol; PMF: Peptide Mass Fingerprint.

## Competing interests

The authors declare that they have no competing interests.

## Authors’ contributions

LD conceived and designed the experiment; LD and Sara Pasella carried out the 2D-PAGE experiments, the interpretation of the data and preparation of the manuscript. JV, GC, CF and GB had contributed to the manuscript preparation. AB and Sara Pasella collected and classified the plasma samples. AB, Sara Pinna, EC participated in the 2D-PAGE experiments and gel bioinformatic analyses. CC, AZ, SS helped with the data analysis. All authors read and approved the final manuscript.

## Supplementary Material

Additional file 1: Table S1 The matched peptides sequences of each protein spots identified through MALDI-TOF MS. Click here for file

Additional file 2: Table S2 The matched peptides sequences of each protein spots identified through LC- MS/MS. Click here for file
