# Supplementary material for: Pre-analytical stability of the plasma proteomes based on the storage temperature
Source: Proteome Sci. 2013 Mar 21;11:10. doi: 10.1186/1477-5956-11-10 (PMC3658880; doi:10.1186/1477-5956-11-10)
Supplement: Additional file 1: Table S1 — The matched peptides sequences of each protein spots identified through MALDI-TOF MS. [file 1477-5956-11-10-S1.pdf]

Supplementary Table S1

The matched peptides sequences of each protein spots identified through MALDI-TOF MS

Spot [a]	Identified protein	Acc. no. [b]	Sequence [c]
**1**	Vitamin D-binding protein	P02774	K.EFSHLGKEDFTSLSLVLYSR.K<br>R.KFPSGTFEQVSQLVK.E<br>K.EVVSLTEACCAEGADPDCYDTR.T<br>K.SCESNSPFPVHPGTAECCTKEGLER.K<br>K.HQPQEFPTYVEPTNDEICEAFR.K<br>K.SYLSMVGSCCTSASPTVCFLK.E Oxidation (M)<br>K.HLSLLTTLSNR.V<br>K.VMDKYTFELSR.R Oxidation (M)<br>R.RTHLPEVFLSK.V<br>K.SLGECCDVEDSTTCFNAK.G<br>R.SDFASNCCSINSPPLYCDSEIDAELKNIL.-
**2**	Serum albumin	P02768	R.DAHKSEVAHR.F<br>R.FKDLGEENFK.A<br>R.LVRPEVDVMCTAFHDNEETFLKK.Y + Oxidation (M)<br>K.YLYEIAR.R<br>R.AFKAWAVAR.L<br>K.VHTECCHGDLLECADDRADLAK.Y<br>R.RHPDYSVVLLLR.L<br>K.VFDEFKPLVEEPQNLIK.Q<br>K.QNCELFEQLGEYKFQNALLVR.Y<br>K.FQNALLVR.Y<br>K.RMPCAEDYLSVVLNQLCVLHEK.T + Oxidation (M)<br>K.EFNAETFTFHADICTLSEKER.Q
**3**	Serum albumin	P02768	R.FKDLGEENFK.A<br>K.LVNEVTEFAK.T<br>R.LVRPEVDVMCTAFHDNEETFLKK.Y Oxidation (M)<br>K.YLYEIAR.R<br>R.HPYFYAPELLFFAKR.Y<br>K.AAFTECCQAADKAACLLPK.L<br>R.AFKAWAVAR.L<br>K.LVTDLTKVHTECCHGDLLECADDR.A<br>K.YICENQDSISSKLK.E<br>K.LKECCEKPLLEK.S<br>K.SHCIAEVENDEMPADLPSLAADFVESK.D Oxidation (M)<br>K.SHCIAEVENDEMPADLPSLAADFVESKDVCK.N Oxidation (M)<br>R.RHPDYSVVLLLR.L<br>K.QNCELFEQLGEYKFQNALLVR.Y Gln->pyro-Glu (N-term Q)<br>K.QNCELFEQLGEYKFQNALLVR.Y<br>R.RPCFSALEVDETYVPK.E<br>K.EFNAETFTFHADICTLSEKER.Q<br>K.AVMDDFAAFVEK.C Oxidation (M)

Spot [a]	Identified protein	Acc. no. [b]	Sequence [c]
**4**	Serum albumin	P02768	R.FKDLGEENFK.A<br>K.LVNEVTEFAK.T<br>R.HPYFYAPELLFFAKR.Y<br>R.YKAAFTECCQAADK.A<br>K.AAFTECCQAADKAACLLPK.L<br>K.VHTECCHGDLLECADDR.A<br>K.VHTECCHGDLLECADDRADLAK.Y<br>K.LKECCEKPLLEK.S<br>K.SHCIAEVENDEMPADLPSLAADFVESK.D + Oxidation (M)<br>K.DVFLGMFLYEYAR.R<br>R.RHPDYSVVLLLR.L<br>K.TYETTLEKCCAAADPHECYAK.V<br>K.CCAAADPHECYAK.V<br>K.VFDEFKPLVEEPQNLIK.Q<br>K.QNCELFEQLGEYKFQNALLVR.Y<br>K.KVPQVSTPTLVEVSR.N<br>K.VPQVSTPTLVEVSR.N<br>R.RPCFSALEVDETYVPK.E<br>K.EFNAETFTFHADICTLSEKER.Q<br>K.AVMDDFAAFVEK.C<br>K.AVMDDFAAFVEK.C + Oxidation (M)
**5**	Alpha-1-antitrypsin	P01009	K.ITPNLAEFAFSLYR.Q<br>R.TLNQPDSQLQLTTGNGLFLSEGLK.L<br>K.LYHSEAFTVNFGDTEEAK.K<br>K.LYHSEAFTVNFGDTEEAKK.Q<br>K.RLGMFNIQHCK.K Oxidation (M)<br>R.LGMFNIQHCK.K Oxidation (M)<br>R.LGMFNIQHCKK.L Oxidation (M)<br>K.FLENEDRR.S<br>K.VFSNGADLSGVTEEAPLK.L<br>K.VFSNGADLSGVTEEAPLKLSK.A<br>K.AVLTIDEKGTEAAGAMFLEAIPMSIPPEVK.F 2 Oxidation (M)<br>K.GTEAAGAMFLEAIPMSIPPEVK.F 2 Oxidation (M)<br>K.FNKPFVFLMIEQNTK.S Oxidation (M)
**6**	Alpha-1-antitrypsin	P01009	K.ITPNLAEFAFSLYR.Q<br>K.LYHSEAFTVNFGDTEEAKK.Q<br>K.DTEEEDFHVDQVTTVK.V<br>K.RLGMFNIQHCK.K Oxidation (M)<br>R.LGMFNIQHCK.K Oxidation (M)<br>K.FLENEDRR.S<br>K.VFSNGADLSGVTEEAPLKLSK.A<br>K.AVLTIDEKGTEAAGAMFLEAIPMSIPPEVK.F 2 Oxidation (M)<br>K.GTEAAGAMFLEAIPMSIPPEVK.F 2 Oxidation (M)<br>K.FNKPFVFLMIEQNTK.S Oxidation (M)

Spot [a]	Identified protein	Acc. no. [b]	Sequence [c]
7	Fibrinogen beta chain	P02675	K.HQLYIDETVNSNIPTNLR.V<br>R.TPCTVSCNIPVVSGKECEEIIR.K<br>R.KGGETSEMYLIQPDSSVKPYR.V + Oxidation (M)<br>K.GGETSEMYLIQPDSSVKPYR.V<br>K.AHYGGFTVQNEANKYQISVNK.Y<br>R.YYWGGQYTWDMAK.H + Oxidation (M)<br>K.IRPFFPQQ.-
8	Fibrinogen beta chain	P02675	K.REEAPSLRPAPPPISGGGYR.A<br>R.EEAPSLRPAPPPISGGGYR.A<br>R.NSVDELNNNVEAVSQTSSSSFQYMYLLK.D Oxidation (M)<br>K.QVKDNENVVNEYSSELEK.H<br>K.DNENVVNEYSSELEKHQLYIDETVNSNIPTNLR.V<br>R.KGGETSEMYLIQPDSSVKPYR.V<br>K.GGETSEMYLIQPDSSVKPYR.V<br>K.GGETSEMYLIQPDSSVKPYR.V Oxidation (M)<br>K.AHYGGFTVQNEANKYQISVNK.Y<br>K.IRPFFPQQ.-
9	Fibrinogen gamma chain	P02679	R.DNCCILDER.F<br>R.FGSYCPTTCGIADFLSTYQTK.V<br>K.AIQLTYNPDESSKPNMIDAATLK.S Oxidation (M)<br>R.YLQEIYNSNNQK.I<br>K.VAQLEAQCQEPCKDTVQIHDITGK.D<br>K.QSGLYFIKPLK.A Gln->pyro-Glu (N-term Q)<br>K.QSGLYFIKPLK.A<br>K.ANQQFLVYCEIDGSGNGWTVFQK.R<br>K.ANQQFLVYCEIDGSGNGWTVFQKR.L<br>K.EGFGHLSPTGTTEFWLGNEK.I<br>K.IHLISTQSAIPYALR.V<br>K.ASTPNGYDNGIIWATWK.T<br>K.IIPFNRLTIGEGQQHHLGGAK.Q<br>R.LTIGEGQQHHLGGAK.Q
10	Fibrinogen gamma chain	P02679	R.FGSYCPTTCGIADFLSTYQTK.V<br>K.AIQLTYNPDESSKPNMIDAATLK.S Oxidation (M)<br>R.YLQEIYNSNNQK.I<br>K.VAQLEAQCQEPCKDTVQIHDITGK.D<br>K.QSGLYFIKPLK.A Gln->pyro-Glu (N-term Q)<br>K.QSGLYFIKPLK.A<br>K.ANQQFLVYCEIDGSGNGWTVFQK.R<br>K.ANQQFLVYCEIDGSGNGWTVFQKR.L<br>K.EGFGHLSPTGTTEFWLGNEK.I<br>K.IHLISTQSAIPYALR.V<br>K.CHAGHLNGVYYQGGTYSK.A

Spot [a]	Identified protein	Acc. no. [b]	Sequence [c]
			K.ASTPNGYDNGIIWATWK.T<br>K.IIPFNRLTIGEGQQHHLGGAK.Q<br>R.LTIGEGQQHHLGGAK.Q
**11**	Fibrinogen gamma chain	P02679	R.DNCCILDER.F<br>R.FGSYCPTTCGIADFLSTYQTK.V<br>R.YLQEIYNSNNQK.I<br>K.VAQLEAQCQEPCKDTVQIHDITGK.D<br>K.QSGLYFIKPLK.A Gln->pyro-Glu (N-term Q)<br>K.QSGLYFIKPLK.A<br>K.ANQQFLVYCEIDGSGNGWTVFQK.R<br>K.ANQQFLVYCEIDGSGNGWTVFQKR.L<br>K.NWIQYKEGFGHLSPTGTTEFWLGNEK.I<br>K.EGFGHLSPTGTTEFWLGNEK.I<br>K.IHLISTQSAIPYALR.V<br>K.ASTPNGYDNGIIWATWK.T<br>K.IIPFNRLTIGEGQQHHLGGAK.Q<br>R.LTIGEGQQHHLGGAK.Q
**12**	Fibrinogen gamma chain	P02679	R.DNCCILDER.F<br>R.FGSYCPTTCGIADFLSTYQTK.V<br>K.AIQLTYNPDESSKPNMIDAATLK.S Oxidation (M)<br>R.YLQEIYNSNNQK.I<br>K.VAQLEAQCQEPCKDTVQIHDITGK.D<br>K.QSGLYFIKPLK.A Gln->pyro-Glu (N-term Q)<br>K.QSGLYFIKPLK.A<br>K.ANQQFLVYCEIDGSGNGWTVFQK.R<br>K.ANQQFLVYCEIDGSGNGWTVFQKR.L<br>K.EGFGHLSPTGTTEFWLGNEK.I<br>K.IHLISTQSAIPYALR.V<br>K.ASTPNGYDNGIIWATWK.T<br>K.IIPFNRLTIGEGQQHHLGGAK.Q<br>R.LTIGEGQQHHLGGAK.Q

Spot [a]	Identified protein	Acc. no. [b]	Sequence [c]
**13**	Serotransferrin	P02787	R.WCAVSEHEATK.C<br>R.WCAVSEHEATKCQSFR.D<br>K.CQSFRDHMK.S Oxidation (M)<br>K.SVIPSDGPSVACVK.K<br>K.KASYLDCIR.A<br>K.ASYLDCIR.A<br>K.KDSGFQMNQLR.G<br>K.KDSGFQMNQLR.G Oxidation (M)<br>K.DSGFQMNQLR.G Oxidation (M)<br>R.SAGWNIPIGLLYCDLPEPR.K<br>K.HSTIFENLANKADR.D<br>K.ADRDQYELLCLDNTR.K<br>R.KPVDEYKDCHLAQVPSHTVVAR.S<br>K.DLLFKDSAHGFLK.V<br>R.MDAKMYLGYEYVTAIR.N Oxidation (M)<br>K.MYLGYEYVTAIR.N<br>R.NLREGTCPEAPTDECKPVK.W<br>K.WCALSHHER.L<br>K.SASDLTWDNLKGK.K<br>K.INHCRFDEFFSEGCAPGSK.K<br>K.CDEWSVNSVGKIECVSAETTEDCIAK.I<br>K.SASDLTWDNLK.G<br>R.FDEFFSEGCAPGSKK.D<br>K.LCMGSGLNLCEPNNK.E Oxidation (M)<br>K.LCMGSGLNLCEPNNKEGYYGYTGAFR.C<br>K.EGYYGYTGAFR.C<br>K.EGYYGYTGAFRCLVEK.G<br>R.CLVEKGDVAFVK.H<br>K.HQTVPQNTGGK.N<br>K.HQTVPQNTGGKNPDPWAK.N<br>K.NLNEKDYELLCLDGTR.K<br>R.KPVEEYANCHLAR.A<br>R.APNHAVVTR.K<br>K.DLLFRDDTVCLAK.L<br>K.LHDRNTYEK.Y<br>R.NTYEKYLGEEYVK.A<br>K.YLGEEYVK.A<br>R.KCSTSSLLEACTFR.R<br>K.CSTSSLLEACTFR.R<br>K.CSTSSLLEACTFRRP.-

Spot [a]	Identified protein	Acc. no. [b]	Sequence [c]
**14**	Serotransferrin	P02787	K.DSGFQMNQLR.G<br>R.SAGWNIPIGLLYCDLPEPR.K<br>R.KPVDEYKDCHLAQVPSHTVVAR.S<br>R.MDAKMYLGYEYVTAIR.N + Oxidation (M)<br>K.MYLGYEYVTAIR.N<br>K.MYLGYEYVTAIR.N + Oxidation (M)<br>K.INHCRFDEFFSEGCAPGSK.K<br>K.LCMGSGLNLCEPNNKEGYYGYTGAFR.C + Oxidation (M)<br>K.NLNEKDYELLCLDGTR.K<br>R.KPVEEYANCHLAR.A
**15**	Serotransferrin	P02787	K.KASYLDCIR.A<br>R.SAGWNIPIGLLYCDLPEPR.K<br>K.ADRDQYELLCLDNTR.K<br>R.KPVDEYKDCHLAQVPSHTVVAR.S<br>R.MDAKMYLGYEYVTAIR.N + Oxidation (M)<br>K.MYLGYEYVTAIR.N<br>K.MYLGYEYVTAIR.N + Oxidation (M)<br>R.NLREGTCPEAPTDECKPVK.W<br>R.EGTCPEAPTDECKPVKWCALSHHER.L<br>K.WCALSHHER.L<br>K.CDEWSVNSVGKIECVSAETTEDCIAK.I<br>K.INHCRFDEFFSEGCAPGSK.K<br>R.FDEFFSEGCAPGSK.K<br>K.EGYYGYTGAFR.C<br>K.NLNEKDYELLCLDGTR.K<br>R.KPVEEYANCHLAR.A<br>K.DLLFRDDTVCLAK.L<br>R.KCSTSSLLEACTFR.R<br>K.CSTSSLLEACTFR.R<br>K.CSTSSLLEACTFRRP.-
**16**	Apolipoprotein A-I	P02647	K.DSGRDYVSQFEGSALGK.Q<br>K.VQPYLDDFQK.K<br>K.VQPYLDDFQKK.W<br>K.WQEEMELYR.Q Oxidation (M)<br>K.VEPLRAELQEGAR.Q<br>R.THLAPYSDELRQR.L<br>K.VSFLSALEEYTK.K

Spot [a]	Identified protein	Acc. no. [b]	Sequence [c]
**17**	Apolipoprotein-E	P02649	R.QQTEWQSGQR.W<br>R.QQTEWQSGQRWELALGR.F<br>R.FWDYLR.W<br>R.WVQTLSEQVQEELLSSQVTQELR.A<br>R.ALMDETMKELK.A<br>K.AYKSELEEQLTPVAEETR.A<br>R.LSKELQAAQAR.L<br>R.GEVQAMLGQSTEELRVR.L Oxidation (M)<br>R.LASHLRK.L<br>R.LAVYQAGAR.E<br>R.LGPLVEQGR.V<br>R.AATVGSLAGQPLQER.A<br>R.AKLEEQAQQIR.L<br>R.LQAEAFQAR.L<br>R.LKSWFEPLVEDMQR.Q<br>R.LKSWFEPLVEDMQR.Q Oxidation (M)<br>K.SWFEPLVEDMQR.Q Oxidation (M)
**18**	Haptoglobin	P00738	K.QWINKAVGDK.L<br>R.VMPICLPSKDYAEVGR.V<br>R.VMPICLPSKDYAEVGR.V + Oxidation (M)<br>K.FTDHLKYVMLPVADQDQCIR.H<br>K.YVMLPVADQDQCIR.H<br>K.YVMLPVADQDQCIR.H + Oxidation (M)<br>K.SPVGVQPILNEHTFCAGMSK.Y<br>K.SPVGVQPILNEHTFCAGMSK.Y + Oxidation (M)<br>K.SCAVAEYGVYVK.V
**19**	Haptoglobin	P00738	K.QWINKAVGDK.L<br>K.DIAPTLTLYVGKK.Q<br>R.VMPICLPSKDYAEVGR.V<br>R.VMPICLPSKDYAEVGR.V Oxidation (M)<br>R.VGYVSGWGR.N<br>R.NANFKFTDHLK.Y<br>K.YVMLPVADQDQCIR.H Oxidation (M)<br>K.SPVGVQPILNEHTFCAGMSK.Y<br>K.SPVGVQPILNEHTFCAGMSK.Y Oxidation (M)<br>K.SCAVAEYGVYVK.V

Spot [a]	Identified protein	Acc. no. [b]	Sequence [c]
**20**	Complement factor B	P00751	R.WSGQTAICDNGAGYCSNPGIPIGTR.K<br>K.EAGIPEFYDYDVALIK.L<br>K.YGQTIRPICLPCTEGTTR.A<br>R.FLCTGGVSPYADPNTCR.G

[a] Spot ID represents the protein spot number on the 2-DE gel image.

[b] Accession numbers of matched proteins according to the SwissProt database.

[c] The sequence of matched peptides.
