# Supplementary material for: Pre-analytical stability of the plasma proteomes based on the storage temperature
Source: Proteome Sci. 2013 Mar 21;11:10. doi: 10.1186/1477-5956-11-10 (PMC3658880; doi:10.1186/1477-5956-11-10)
Supplement: Additional file 2: Table S2 — The matched peptides sequences of each protein spots identified through LC- MS/MS. [file 1477-5956-11-10-S2.pdf]

Supplementary Table S2

The matched peptides sequences of each protein spots identified through LC- MS/MS

Spot [a]	Identified protein	Acc. no. [b]	Sequence [c]
**1**	Vitamin D-binding protein	P02774	R.LSNLIK.L<br>K.VLEPTLK.S<br>K.LCDNLSTK.N<br>R.THLPEVFLSK.V<br>R.THLPEVFLSK.V<br>K.LPDATPKELAK.L<br>R.VCSQYAAYGEK.K<br>R.RTHLPEVFLSK.V<br>K.VMDKYTFELSR.R + Oxidation (M)<br>K.VMDKYTFELSR.R + Oxidation (M)<br>K.VMDKYTFELSR.R + Oxidation (M)<br>K.NSKFEDCCQEK.T<br>K.SLGECCDVEDSTTCFNAK.G<br>K.SLGECCDVEDSTTCFNAK.G<br>K.SLGECCDVEDSTTCFNAK.G<br>K.SLGECCDVEDSTTCFNAK.G<br>K.SYLSMVGSCCTSASPTVCFLK.E + Oxidation (M)<br>K.EVVSLTEACCAEGADPDCYDTR.T<br>K.EVVSLTEACCAEGADPDCYDTR.T<br>K.HQPQEFPTYVEPTNDEICEAFR.K<br>K.LAQKVPTADLEDVLPLAEDITNILSK.C<br>K.VLEPTLKSLGECCDVEDSTTCFNAK.G<br>R.SDFASNCCSINSPPLYCDSEIDAELK.N<br>R.SDFASNCCSINSPPLYCDSEIDAELK.N
**13**	Serotransferrin	P02787	R.APNHAVVTR.K<br>R.APNHAVVTR.K<br>K.ASYLDCIR.A<br>K.ASYLDCIR.A<br>R.APNHAVVTRK.D<br>K.KASYLDCIR.A<br>K.KASYLDCIR.A<br>K.KASYLDCIR.A<br>K.DSGFQMNQLR.G<br>K.DSGFQMNQLR.G<br>K.DSGFQMNQLR.G + Oxidation (M)<br>K.EGYYGYTGAFR.C<br>R.WCAVSEHEATK.C<br>R.WCAVSEHEATK.C<br>K.KDSGFQMNQLR.G<br>R.CLVEKGDVAFVK.H<br>R.CLVEKGDVAFVK.H<br>K.CLKDGAGDVAFVK.H<br>K.CLKDGAGDVAFVK.H<br>K.SVIPSDGPSVACVK.K<br>K.SVIPSDGPSVACVK.K<br>K.SASDLTWDNLKGK.K<br>K.SASDLTWDNLKGK.K<br>K.MYLGYEYVTAIR.N<br>K.MYLGYEYVTAIR.N<br>K.MYLGYEYVTAIR.N + Oxidation (M)

Spot [a]	Identified protein	Acc. no. [b]	Sequence [c]
			K.MYLGYEYVTAIR.N + Oxidation (M)<br>K.CSTSSLLEACTFR.R<br>K.SVIPSDGPSVACVKK.A<br>K.SVIPSDGPSVACVKK.A<br>K.SVIPSDGPSVACVKK.A<br>K.DLLFRDDTVCLAK.L<br>R.FDEFFSEGCAPGSK.K<br>R.KPVEEYANCHLAR.A<br>K.YLGEEYVKAVGNLR.K<br>K.CSTSSLLEACTFRRP.-<br>K.CSTSSLLEACTFRRP.-<br>K.CSTSSLLEACTFRRP.-<br>R.MDAKMYLGYEYVTAIR.N + Oxidation (M)<br>K.NLNEKDYELLCLDGTR.K<br>K.IMNGEADAMSLDGGFVYIAGK.C + 2 Oxidation (M)<br>R.NLREGTCPEAPTDECKPVK.W<br>R.KPVDEYKDCHLAQVPSHTVVAR.S<br>K.LCMGSGLNLCEPNNKEGYYGYTGAFR.C + Oxidation (M)<br>K.IECVSAETTEDCIAKIMNGEADAMSLDGGFVYIAGK.C
**17**	Apolipoprotein E	P02649	R.GLSAIRER.L<br>R.LAVYQAGAR.E<br>R.LGPLVEQGR.V<br>R.LQAEAFQAR.L<br>R.LSKELQAAQAR.L<br>R.LSKELQAAQAR.L<br>R.LGADMEDVCGR.L<br>R.LGPLVEQGRVR.A<br>R.LGADMEDVCGR.L + Oxidation (M)<br>R.ALMDETMKELK.A + 2 Oxidation (M)<br>R.LAVYQAGAREGAER.G<br>R.AATVGSLAGQPLQER.A<br>R.AATVGSLAGQPLQER.A<br>K.VQAAVGTSAAPVPSDNH.-<br>K.AYKSELEEQLTPVAEETR.A

[a] Spot ID represents the protein spot number on the 2-DE gel image.

[b] Accession numbers of matched proteins according to the SwissProt database.

[c] The sequence of matched peptides.
